# Construction and evaluation of a diagnostic model for metabolic dysfunction-associated steatotic liver disease based on advanced glycation end products and their receptors

**DOI:** 10.3389/fmed.2025.1539708

**Published:** 2025-03-28

**Authors:** Xiao Cao, Xiaohua Xiao, Peipei Jiang, Nian Fu

**Affiliations:** Department of Gastroenterology, Hunan Provincial Clinical Research Center for Metabolic Associated Fatty Liver Diseases, The Affiliated Nanhua Hospital, Hengyang Medical School, University of South China, Hengyang, Hunan, China

**Keywords:** MASLD, non-obese, advanced glycation end products, diagnostic model, esRAGE

## Abstract

**Background:**

Effective biomarkers for the diagnosis of metabolic dysfunction-associated steatotic liver disease (MASLD) remain limited. This study aims to evaluate the potential of advanced glycation end products (AGEs) and their endogenous secretory receptor (esRAGE) as non-invasive biomarkers for diagnosing MASLD, to explore differences between obese and non-obese MASLD patients, and to develop a novel diagnostic model based on these biomarkers.

**Methods:**

This study enrolled 341 participants, including 246 MASLD patients (118 non-obese, 128 obese) and 95 healthy controls. Serum AGEs and esRAGE levels were measured by ELISA. Key predictors were identified using the Lasso algorithm, and a diagnostic model was developed with logistic regression and visualized as nomograms. Diagnostic accuracy and utility were evaluated through the area under the curve (AUC), bootstrap validation, calibration curves, and decision curve analysis (DCA).

**Results:**

Serum AGEs and esRAGE levels were significantly higher in MASLD patients compared to controls. Moreover, obese MASLD patients had higher esRAGE levels than non-obese ones, but no significant difference in AGEs levels was found. A diagnostic model incorporating age, WC, BMI, ALT, TG, HDL, AGEs, and esRAGE achieved an AUC of 0.963, with 94.3% sensitivity and 85.3% specificity. The AUC for bootstrap internal validation was 0.963 (95% CI: 0.944–0.982). Calibration curves showed strong predictive accuracy, and DCA demonstrated high net clinical benefit.

**Conclusion:**

Serum AGEs and esRAGE serve as non-invasive biomarkers for distinguishing MASLD patients. We developed and validated diagnostic models for MASLD, offering valuable tools to identify at-risk populations and improve prevention and treatment strategies.

## 1 Introduction

Metabolic dysfunction-associated steatotic liver disease (MASLD) is the most widespread chronic liver disorder globally, impacting more than one-third of adults (1). Projections indicate that by 2040, the prevalence could exceed 50% of the adult population worldwide (2). Although liver biopsy remains the gold standard for diagnosing MASLD, enabling the classification of hepatic steatosis, fibrosis, and inflammation (3), it is invasive, carries risks of complications, and is limited by sampling variability and significant interobserver variability in pathology interpretation (4, 5). In this context, the development of simple, noninvasive tools for routine clinical use, including various serum-based scoring systems, has been proposed as viable alternatives, such as the fatty liver index (FLI) and the hepatic steatosis index (HSI) (6–9). While models like FLI and HSI are commonly used in MASLD screening, they have notable limitations, primarily because they rely heavily on traditional metabolic parameters (e.g., BMI, lipid levels, liver function indices) and are predominantly based on populations from Western countries (10, 11). Approximately 40% of the global MASLD population is categorized as non-obese (12). Patients with MASLD who are obese and those who are not have notable variations in their clinical and metabolic characteristics (13). This presents challenges in diagnosing MASLD using scoring systems based solely on clinical characteristics and basic serum markers. Therefore, identifying serum biomarkers with high discriminatory power for both non-obese and obese MASLD is essential. The “multiple- hit” hypothesis has recently emerged, proposing that various factors act simultaneously and synergistically to induce metabolic and molecular changes, driving the onset and progression of MASLD (14, 15). Within this framework, several studies have highlighted the significant role of advanced glycation end products (AGEs) in the pathogenesis of MASLD (16–18).

AGEs form through a non-enzymatic reaction between amine groups in proteins, lipids, nucleic acids, and carbonyl groups in reducing sugars (19). The receptor for advanced glycation end products (RAGE) is a transmembrane receptor that recognizes products of non-enzymatic glycation and protein oxidation (20). When AGEs bind to RAGE, it triggers various intracellular signaling pathways that enhance cytokine production. This cascade eventually results in lipid buildup in the liver, promoting steatosis and the development of MASLD (18, 21–23). Several studies suggest that AGEs may serve as potential plasma biomarkers for diagnosing MASLD (24, 25). The endogenous secretory receptor for advanced glycation end products (esRAGE) is a C-truncated splice variant of RAGE that functions as a decoy receptor for RAGE ligands, modulating RAGE signaling (26). Levels of circulating esRAGE can be utilized as a measure of RAGE generation in tissues (27), as well as a reaction to reduce RAGE-induced tissue damage (20, 26, 28). Thus, the aim of this study was to investigate the potential of AGEs and esRAGE as non-invasive biomarkers for MASLD, and to develop and evaluate a novel diagnostic model based on these biomarkers.

## 2 Materials and methods

### 2.1 Participants

Participants for this cross-sectional research were recruited from Nanhua Hospital, which is connected with Nanhua University, between January and September 2024. There were 246 patients with a diagnosis of MASLD (118 with non-obese MASLD and 128 with obese MASLD) and 95 healthy controls among the 341 people that were included. The MASLD diagnostic criteria were based on the Multi-Society Delphi Consensus Statement on New Nomenclature for Fatty Liver Disease (29). Inclusion criteria consisted of participants aged 18–75 years, with an ultrasound-confirmed diagnosis of fatty liver, exclusion of excessive alcohol consumption (≥210 g/week for men and ≥ 140 g/week for women), exclusion of other causes of fatty liver, and the presence of at least one component of metabolic syndrome. Patients with chronic inflammatory conditions were excluded. Cases with missing data were excluded from the analysis. MASLD patients were split into two groups: those who were non-obesity (BMI < 25 kg/m^2^) and those who were obese (BMI ≥ 25 kg/m^2^). The study was authorized by the Nanhua Hospital Ethics Committee at Nanhua University (approval code: 2024-Ky-232), and all participants gave signed informed consent. The research complied with the 1975 Declaration of Helsinki’s ethical guidelines.

### 2.2 Clinical and laboratory data collection

Body Mass Index (BMI), waist circumference (WC), and systolic blood pressure (SBP) from the right arm were among the anthropometric measurements that were gathered. Following a period of fasting lasting 12 h, blood samples were drawn. Hematological parameters, including white blood cells (WBC), neutrophils, monocytes, hemoglobin, and platelets, were assessed using an automatic blood cell analyzer at the Nanhua Hospital Laboratory, Nanhua University. Biochemical parameters, including albumin, fasting blood glucose (FPG), direct bilirubin (DB), bile acids (BA), alanine aminotransferase (ALT), aspartate aminotransferase (AST), total bilirubin (TB), uric acid (UA), urea nitrogen (BUN), creatinine, total cholesterol (TC), triglycerides (TG), high-density lipoprotein (HDL), low-density lipoprotein (LDL), sodium, and potassium, were measured using an automated biochemical analyzer.

### 2.3 Plasma AGEs and esRAGE

Serum samples were separated from fasting patients and kept at −80°C before analysis. Mlbio’s ELISA kit measured serum AGE and esRAGE levels. In these assays, undiluted serum samples were utilized, and the measurements were conducted following the manufacturer’s guidelines.

### 2.4 Statistical analysis

The quantitative data were non-normally distributed, as determined by the Shapiro-Wilk test. Quantitative data were presented as medians with interquartile ranges (P25, P75) and analyzed using the Mann-Whitney U test, whereas qualitative data were compared using the chi-square test. The correlation between AGEs, esRAGE, and clinical indicators were looked at using Spearman’s correlation coefficient. R 4.4.1 and SPSS version 28.0 were used for all statistics studies.

The predictors selected through the Lasso method for clinical modeling were first subjected to univariate logistic regression analysis to identify clinically significant variables. Construction of the final model using multiple logistic regression analysis. The area under the receiver operating characteristic (ROC) curve (AUC) was used to measure performance. With the help of the “caret” R package, internal validation was carried out using the 1,000-iteration Bootstrap resampling approach. ROC curves were constructed with the help of the “pROC” R package, and a nomogram was made with the help of the “rms” R package. The “rms” and “ResourceSelection” R packages were used to plot calibration curves. The “rmda” R packages was used to perform decision curve analysis (DCA), which evaluated the diagnostic model’s clinical usefulness. Common MASLD indices, including his (30), Korea National Health and Nutrition Examination Survey-derived nonalcoholic fatty liver disease score (K-NAFLD) (31), the NAFL Screening Score (NSS) (32), and the NAFLD Ridge Score (33), were calculated according to the methods described in the original publications.

## 3 Results

### 3.1 Association between AGEs and esRAGE and MASLD

**FIGURE 1:**
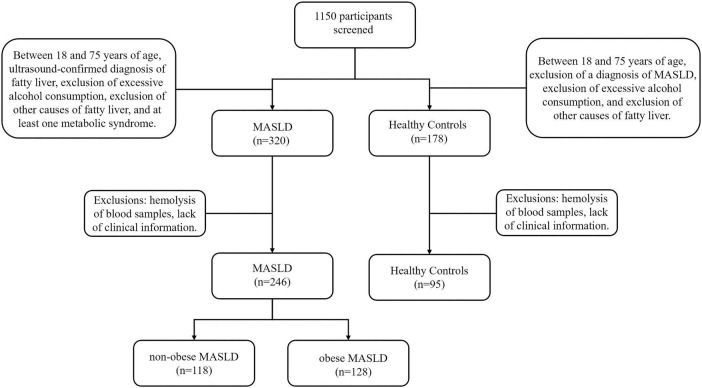
Participant selection flowchart.

Following stringent screening and exclusion standards, 341 people in all were included in the study: 246 patients with MASLD, of whom 118 were categorized as non-obese MASLD and 128 as obese MASLD, and 95 normal controls (Figure 1). The anthropometric and laboratory parameters of these participants are summarized in Table 1. The cohort included 159 men (46.6%) and 182 females (53.4%). The MASLD group had a considerably larger proportion of males than the normal control group (*P* < 0.001). The cohort’s median age was 54 years, and patients with MASLD were notably older than the normal controls (*P* < 0.001). Anthropometric measures showed that the MASLD group had substantially greater WC, BMI, and SBP than the controls (*P* < 0.001). Similarly, laboratory findings showed significantly higher levels of WBC, neutrophils, monocytes, hemoglobin, ALT, AST, UA, BUN, TC, TG, LDL, and FBG in MASLD patients (*P* < 0.05). Conversely, there was a substantial decrease in HDL values in the MASLD group (*P* < 0.05). The groups’ levels of platelets, albumin, TB, DB, BA, creatinine, sodium, and potassium did not differ much.

**TABLE 1:** Characteristics of participants.

Characteristics	Non-MASLD (*n* = 95)	MASLD (*n* = 246)	*P*-value
Sex (male)	23(24.2%)	136(55.3%)	<0.001
Age	47(40–54)	55(49–60)	<0.001
WC	78(75–83)	92(87–97)	<0.001
BMI	21.64(19.22–23.31)	25.25(23.44–28.03)	<0.001
SBP	119(107–127)	130(121–138)	<0.001
WBC	5.41(4.61–5.99)	6.19(5.19–7.64)	<0.001
Neutrophils	3.32(2.65–4.14)	3.9(3.21–5.21)	<0.001
Monocytes	0.35(0.27–0.45)	0.44(0.35–0.54)	<0.001
Hemoglobin	127(119–136)	138(126–149)	0.001
Platelets	215(164–251)	220.5(180–263.25)	0.691
Albumin	45.2(43.5–47.2)	44.65(42.68–47.3)	0.357
ALT	14.1(11.1–17.3)	21.7(16–32.63)	<0.001
AST	19.2(16.5–21.4)	20.85(16.5–26.3)	0.018
TB	12.6(9.2–16.69)	11.9(8.28–16.43)	0.55
DB	3.2(2.5–4.3)	3.1(2.3–4.13)	0.289
BA	3.3(2.0–5.7)	3.8(2.4–6.83)	0.115
UA	250(217–295)	340.5(284.75–417.25)	<0.001
Creatinine	65(56–76)	73.45(61.0–89.0)	0.074
BUN	4.3(3.4–5.17)	5.18(4.0–6.0)	<0.001
TC	4.35(3.76–4.73)	4.73(3.91–5.46)	<0.001
TG	0.99(0.79–1.36)	2.07(1.37–2.82)	<0.001
HDL	1.39(1.2–1.62)	1.03(0.88–1.27)	<0.001
LDL	2.64(2.11–3.07)	3.03(2.32–3.64)	0.001
FBG	5.07(4.79–6.1)	5.77(4.92–7.7)	0.002
Sodium	140.3(138.7–142.4)	140.2(138.6–142.0)	0.398
Potassium	3.95(3.74–4.18)	3.97(3.73–4.26)	0.706
AGEs	9.84(5.94–12.91)	13.53(7.96–18.69)	<0.001
esRAGE	1.9(1.49–2.23)	2.21(1.71–2.74)	<0.001

The data are expressed as either number (percentage) or median (P25–P75). To compare the characteristics between the two groups, the Mann-Whitney U test was used for continuous variables, while the Chi-squared test was employed for categorical variables. WC, Waist Circumference; BMI, Body Mass Index; SBP, Systolic Blood Pressure; WBC, White Blood Cells; FPG, Fasting Plasma Glucose; ALT, Alanine Aminotransferase; AST, Aspartate Aminotransferase; TB, Total Bilirubin; DB, Direct Bilirubin; BA, Bile Acids; UA, Uric Acid; BUN, Urea Nitrogen; TC, Total Cholesterol; TG, Triglycerides; HDL, High-Density Lipoprotein; LDL, Low-Density Lipoprotein; AGEs, Advanced Glycation End Products; esRAGE, Endogenous Secretory Receptor for Advanced Glycation End Products.

**FIGURE 2:**
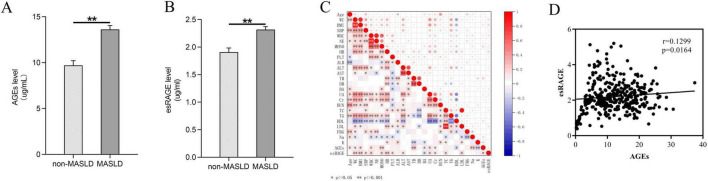
Comparative analysis of AGEs and esRAGE levels in normal controls versus patients with MASLD. **(A)** Patients with MASLD had considerably higher levels of AGEs (*P* < 0.001). **(B)** MASLD patients had considerably higher levels of esRAGE (*P* < 0.001). **(C)** A heatmap depicting the correlation analysis among all candidate predictors showed statistical significance, indicated by asterisks (*) and double asterisks (**), in the correlations between variables. **(D)** Scatterplot of AGEs versus esRAGE, with a positive correlation (*P* = 0.0164).

The distribution of AGEs and esRAGE in normal controls and MASLD patients is illustrated in Figure 2. MASLD patients had considerably greater levels of both AGEs and esRAGE than healthy controls (*P* < 0.001) (Figures 2a,b). To visualize the relationships among the predictor variables, a correlation heatmap was generated, incorporating all continuous predictor variables with the degree of correlation between them indicated on the plot (Figure 2C). The colors in the heatmap indicate the direction of the correlations, with the intensity of the color representing the strength of the correlation. AGEs showed positive correlations with age, WC, BMI, WBC, monocytes, TB, BA, UA, creatinine, sodium, and esRAGE (*P* < 0.05), but showed negative correlations with HDL and potassium (*P* < 0.05). Similarly, esRAGE was positively correlated with WC, BMI, SBP, WBC, monocytes, hemoglobin, platelets, ALT, UA, creatinine, TC, LDL, and AGEs (*P* < 0.05), but negatively correlated with BA. The association between AGEs and esRAGE was analyzed using Spearman’s correlation test, and the resulting scatter plot demonstrated a substantial positive correlation (*r* = 0.1299, *P* = 0.0164) (Figure 2D).

### 3.2 Comparative analysis of AGEs and esRAGE in non-obese and obese MASLD

The anthropometric and laboratory parameters of non-obese and obese MASLD patients are presented in Table 2. The proportion of males among those with obese MASLD (68.7%) was significantly greater than that of males with non-obese MASLD (40.7%) (*P* < 0.001). Additionally, non-obese MASLD patients were substantially older than their obese counterparts (*P* < 0.001). Non-obese MASLD patients had considerably lower BMI and WC levels (*P* < 0.001). Regarding laboratory parameters, serum levels of monocytes, hemoglobin, UA, creatinine, and potassium were significantly elevated in obese MASLD patients (*P* < 0.05). There were no significant differences in WBC, neutrophils, platelets, albumin, ALT, AST, TB, DB, BA, BUN, TC, TG, HDL, LDL, FBG, or sodium levels across groups.

**TABLE 2:** Characteristics of patients with non-obese and obese MASLD.

Characteristics	Non-obese MASLD (*n* = 118)	Obese MASLD (*n* = 128)	*P*-value
Sex (male)	48(40.7%)	88(68.8%)	<0.001
Age	56.5(52–61)	54(45–58)	<0.001
WC	87(82–90)	96(92–100)	<0.001
BMI	23.44(22.12–24.33)	27.76(26.56–29.76)	<0.001
SBP	127(118–139)	131(124–138)	0.098
WBC	6.0(4.89–7.76)	6.36(5.41–7.53)	0.223
Neutrophils	3.84(3.12–5.58)	3.91(3.25–4.93)	0.912
Monocytes	0.41(0.32–0.51)	0.46(0.36–0.57)	0.028
Hemoglobin	135(124–143)	141(128.25–154)	0.001
Platelets	214.5(175.5–257)	225(183.25–265)	0.321
Albumin	45.1(42.9–47.3)	44.3(42.3–47.18)	0.306
ALT	21(15.38–31.1)	22.65(16.7–36.2)	0.073
AST	21.25(16.08–26.93)	10.35(16.78–25.93)	0.922
TB	11.93(8.18–17.23)	11.85(8.31–15.53)	0.368
DB	2.99(2.15–3.95)	3.2(2.34–4.3)	0.155
BA	4.1(2.38–6.73)	3.6(2.43–7.3)	0.977
UA	318(260.75–399)	363.5(304–431.75)	<0.001
Creatinine	68.3(58.9–83.03)	79.7(65.25–95)	<0.001
BUN	5.09(4.08–5.86)	5.18(4.0–6.17)	0.473
TC	4.77(4.06–5.63)	4.66(3.88–5.38)	0.263
TG	2.11(1.31–2.89)	2.01(1.38–2.8)	0.928
HDL	1.08(0.89–1.29)	1.01(0.87–1.25)	0.199
LDL	3.04(2.32–3.75)	3.01(2.32–3.62)	0.749
FBG	5.78(4.94–8.27)	5.78(4.82–7.09)	0.237
Sodium	140.4(138.6–142.0)	139.9(138.53–141.98)	0.72
Potassium	3.95(3.66–4.15)	4.01(3.81–4.3)	0.033
AGEs	13.38(8.91–17.01)	17.9(7.33–20.7)	0.363
esRAGE	2.09(1.64–2.58)	2.28(1.84–2.94)	0.023

The data are expressed as either number (percentage) or median (P25–P75). To compare the characteristics between the two groups, the Mann-Whitney U test was used for continuous variables, while the Chi-squared test was employed for categorical variables. WC, Waist Circumference; BMI, Body Mass Index; SBP, Systolic Blood Pressure; WBC, White Blood Cells; FPG, Fasting Plasma Glucose; ALT, Alanine Aminotransferase; AST, Aspartate Aminotransferase; TB, Total Bilirubin; DB, Direct Bilirubin;, BA, Bile Acids; UA, Uric Acid; BUN, Urea Nitrogen; TC, Total Cholesterol; TG, Triglycerides; HDL, High-Density Lipoprotein; LDL, Low-Density Lipoprotein; AGEs, Advanced Glycation End Products; esRAGE, Endogenous Secretory Receptor for Advanced Glycation End Products.

**FIGURE 3:**
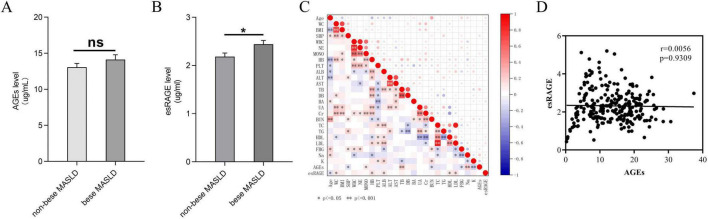
AGEs and esRAGE levels in obese and non-obese MASLD patients are compared. **(A)** No substantial variations in AGE levels were seen between non-obese and obese MASLD patients. **(B)** Patients with MASLD who were obese had elevated levels of esRAGE (*P* < 0.05). **(C)** A heatmap depicting the correlation analysis among all candidate predictors showed statistical significance, indicated by asterisks (*) and double asterisks (**), in the correlations between variables. **(D)** AGEs and esRAGE did not show a statistically significant link, according to Spearman’s correlation analysis.

The distribution of AGEs and esRAGE levels in non-obese and obese MASLD patients is illustrated in Figure 3. Although levels of AGEs elevated in obese MASLD patients compared with non-obese individuals, the difference was not statistically significant (Figure 3A). In contrast, obese MASLD patients had substantially elevated esRAGE levels compared to non-obese patients (*P* < 0.05) (Figure 3B). To illustrate the correlations among all candidate predictor variables in MASLD patients, a correlation heatmap was generated (Figure 3C). The correlation between AGEs and esRAGE was further examined using Spearman’s test, and the resulting scatter plot indicated no significant correlation between AGEs and esRAGE in MASLD patients (Figure 3D).

### 3.3 Diagnostic models based on AGEs and esRAGE

#### 3.3.1 Construction of the model

**FIGURE 4:**
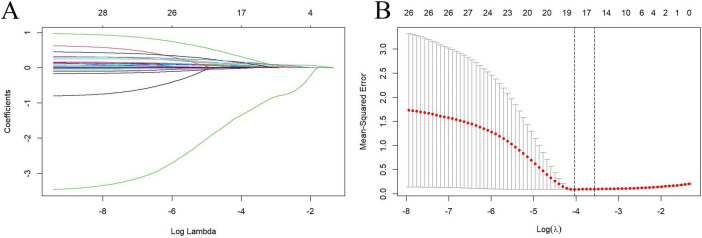
Factor selection was conducted using Lasso binary logistic regression models. **(A)** The logarithm of the lambda (λ) values for the 28 candidate variables in the Lasso model was determined. **(B)** log (Lambda) value that was most appropriate in the LASSO model.

To mitigate the risk of overfitting, we conducted LASSO regression analysis on all candidate predictor variables, generating LASSO coefficient path diagrams (Figure 4A) and LASSO regularized path diagrams (Figure 4B). Based on the optimal λ-value, we retained 18 characteristic variables: age, WC, BMI, SBP, neutrophils, monocytes, ALT, TB, UA, TC, TG, HDL, LDL, FBG, sodium, potassium, AGEs, esRAGE.

Univariate regression analysis of the 18 retained characteristic variables revealed that 15 were significantly associated with MASLD (P < 0.05) (Table 3). The results indicated that the odds ratios (ORs) were as follows: age, 1.089 (1.061–1.117); waist circumference, 1.265 (1.201–1.332); Body Mass Index, 1.769 (1.545–2.025); systolic blood pressure, 1.076 (1.053–1.100); neutrophils, 1.381 (1.146–1.665); monocytes, 50.064 (7.378–339.706); alanine aminotransferase, 1.140 (1.093–1.189); uric acid, 1.015 (1.011–1.020); total cholesterol, 1.583 (1.227–2.042); triglycerides, 4.464 (2.852–6.989); high-density lipoprotein, 0.038 (0.016–0.092); low-density lipoprotein, 1.709 (1.280–2.283); fasting blood glucose, 1.321 (1.127–1.549); AGEs, 1.106 (1.061–1.153); and esRAGE, 1.979 (1.408–2.781). These variables were included in the multivariate regression analysis, which employed a stepwise backward selection method to establish the final model. This resulted in eight characteristic variables retained in the final model: age, 1.146 (1.079–1.218); waist circumference, 1.139 (1.032–1.258); Body Mass Index, 1.381 (1.073–1.777); alanine transaminase, 1.103 (1.042–1.168); triglycerides, 1.522 (1.088–2.129); high-density lipoprotein, 0.201 (0.052–0.773); AGEs, 1.113 (1.027–1.206); and esRAGE, 2.259 (1.154–4.422) (Table 3). The score can be calculated using the following equation:

**TABLE 3:** Table of MASLD risk variables analyzed using univariate and multivariate logistic regression.

Variable	Univariable analysis		Multivariable analysis	
	**OR (95%CI)**	***P*-value**	**OR (95%CI)**	***P*-value**
Age	**1.089(1.061–1.117)**	**<0.001**	**1.146(1.079–1.218)**	**<0.001**
WC	**1.265(1.201–1.332)**	**<0.001**	**1.139(1.032–1.258)**	**0.01**
BMI	**1.769(1.545–2.025)**	**<0.001**	**1.381(1.073–1.777)**	**0.012**
SBP	**1.076(1.053–1.1)**	**<0.001**		
Neutrophils	**1.381(1.146–1.665)**	**0.001**		
Monocytes	**50.064(7.378–339.706)**	**<0.001**		
ALT	**1.14(1.093–1.189)**	**<0.001**	**1.103(1.042–1.168)**	**0.001**
TB	0.98(0.943–1.02)	0.31		
UA	**1.015(1.011–1.02)**	**<0.001**		
TC	**1.583(1.227–2.042)**	**<0.001**		
TG	**4.464(2.852–6.989)**	**<0.001**	**1.522(1.088–2.129)**	**0.014**
HDL	**0.038(0.016–0.092)**	**<0.001**	**0.201(0.052–0.773)**	**0.019**
LDL	**1.709(1.28–2.283)**	**<0.001**		
FBG	**1.321(1.127–1.549)**	**0.001**		
Sodium	0.965(0.899–2.357)	0.331		
Potassium	1.278(0.693–2.357)	0.433		
AGEs	**1.106(1.061–1.153)**	**<0.001**	**1.113(1.027–1.206)**	**0.009**
esRAGE	**1.979(1.408–2.781)**	**<0.001**	**2.259(1.154–4.422)**	**0.017**

Univariate and multivariate logistic regression analyses were conducted to identify risk factors for MASLD. OR: ratio of ratios; Statistical significance is shown by bold values (*P* < 0.05).

MASLD Score = 0.136 × Age (years) + 0.130 × WC (cm) + 0.323 × BMI (kg/m^2^) + 0.098 × ALT (U/L) + 0.420 × TG (mmol/L) − 0.603 × HDL (mmol/L) + 0.107 × AGEs (μg/mL) + 0.815 × esRAGE (μg/mL) − 28.240

#### 3.3.2 Validation of the model

**FIGURE 5:**
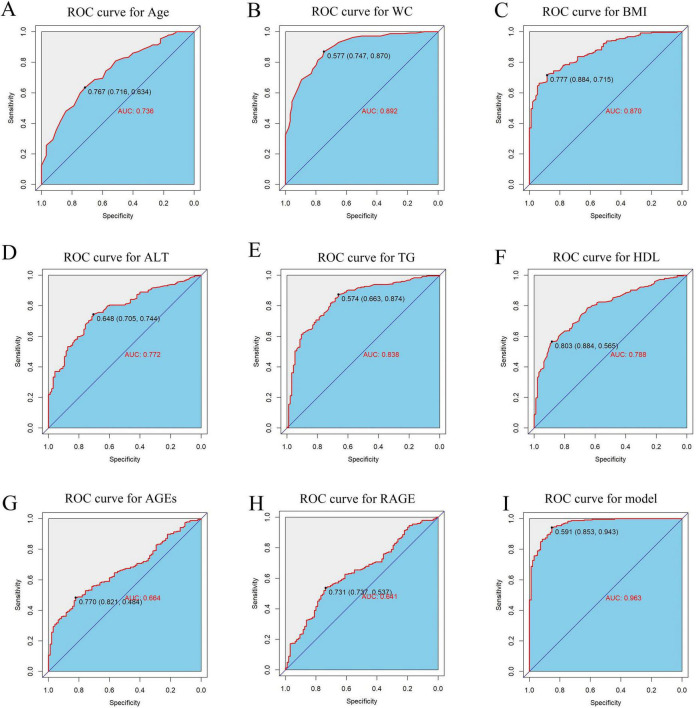
ROC curves for predictors and MASLD diagnostic models. **(A–H)** ROC curves for predictors Age, WC, BMI, ALT, TG, HDL, AGEs, esRAGE models. **(I)** ROC curves for diagnostic models.

**FIGURE 6:**
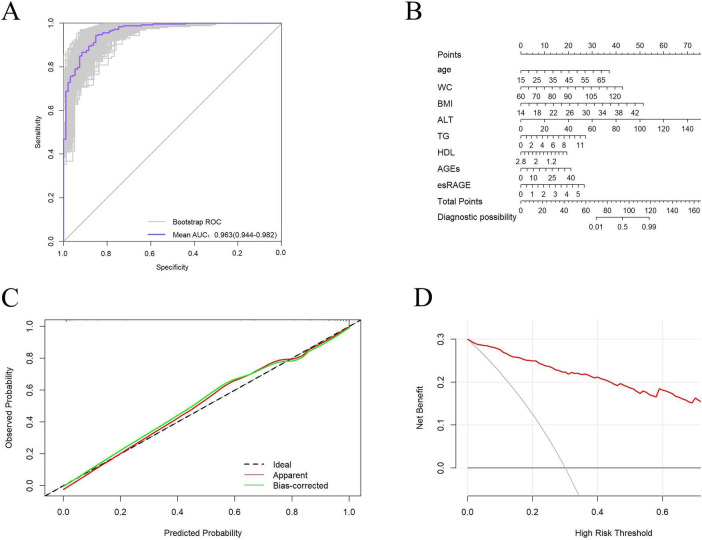
Validation of the diagnostic models. **(A)** Bootstrap internally validated ROC curves. **(B)** Nomogram for predicting MASLD risk classification. **(C)** Model calibration curves. **(D)** When compared to a “all treatment” or “no treatment strategy, DCA demonstrates the net advantage of using the model to diagnose MASLD at different decision thresholds.

Following logistic regression analysis, eight variables were determined to be independent predictors (Table 3). ROC analyses were conducted on these predictors to assess their capability in differentiating MASLD, with results displayed in Figures 5A–H. The area under the curve (AUC) values indicated strong discriminatory performance, demonstrating their accuracy in diagnosing MASLD. The predictive power of the MASLD diagnostic model was significantly higher than that of the univariate clinical model, with an AUC of 0.963 (Figure 5I). In the MASLD model, the sensitivity, specificity, positive predictive value (PPV), negative predictive value (NPV), positive likelihood ratio (PLR), and negative likelihood ratio (NLR) were 94.3, 85.3, 94.6, 84.7, 6.41, and 0.07%, respectively (Table 4). For internal validation, the model was assessed using 1,000 Bootstrap resamplings, and it yielded an AUC of 0.963 (95% CI: 0.944–0.982) (Figure 6A). To enhance diagnostic efficiency, we developed a nomogram incorporating the final variables to estimate the risk of MASLD (Figure 6B). Meanwhile, calibration curves indicated good alignment between predicted and observed probabilities (Figure 6C). Additionally, a decision curve analysis evaluated the model’s utility in clinical decision-making. The model curve, as illustrated in Figure 6D, diverged from the extreme “none” and “all” curves, showing that patients with MASLD could benefit significantly from the diagnostic model in terms of net clinical outcomes. These findings demonstrate that the diagnostic model, which incorporates AGEs and esRAGE, has strong diagnostic efficacy and practical utility.

**TABLE 4:** Diagnostic accuracy of the model.

Models	AUC	Sensitivity (%)	Specificity (%)	PPV (%)	NPV (%)	PLR	NLR	Youden’s index
Age	0.736	63.4	71.6	81	50.2	2.23	0.51	0.350
WC	0.892	87	74.7	86.2	75.9	3.43	0.17	0.617
BMI	0.87	71.5	88.4	92	63.7	6.19	0.32	0.599
ALT	0.772	74.4	70.5	81.5	60.8	2.52	0.36	0.449
TG	0.838	87.4	66.3	82.4	74.3	2.59	0.19	0.537
HDL	0.788	56.5	88.4	89.7	52.7	4.87	0.49	0.449
AGEs	0.664	48.4	82.1	83.2	46.7	2.71	0.63	0.305
esRAGE	0.641	53.7	73.7	79.1	46.2	2.04	0.63	0.274
MASLD Score	0.963	94.3	85.3	94.6	84.7	6.41	0.07	0.796
HSI	0.926	80.5	92.6	95.7	68.1	10.88	0.21	0.731
K-NAFLD	0.914	86.2	80	89.5	75.3	4.31	0.17	0.662
NSS	0.94	82.5	95.8	98	67.9	19.64	0.18	0.783
NAFLD Ridge Score	0.703	62.5	74.7	78	57.4	2.47	0.5	0.372

NPV, negative predictive value; PPV, positive predictive value; NLR, negative likelihood ratio; PLR, positive likelihood ratio.

**FIGURE 7:**
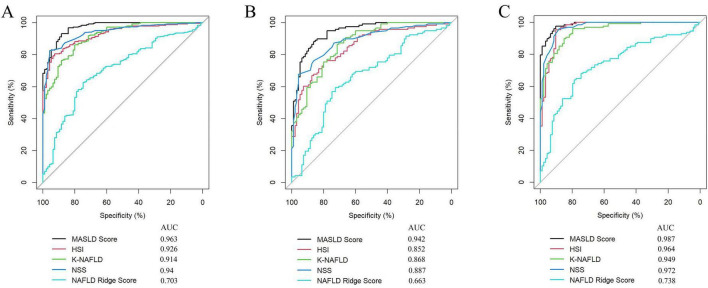
Comparison of the MASLD Score with MASLD-related scoring systems. **(A)** ROC curve analysis of the MASLD score, HSI, K-NAFLD, NSS, and NAFLD Ridge Score in the MASLD cohort. **(B)** Comparison of the diagnostic performance of the models in the non-obese MASLD population. **(C)** Comparison of the diagnostic performance of the models in the obese MASLD population.

The diagnostic performance of five MASLD diagnostic scores (i.e., MASLD Score, HSI, K-NAFLD, NSS, and NAFLD Ridge Score) was systematically evaluated (Table 4). The results indicated that the MASLD Score exhibited superior diagnostic performance compared with the other scoring systems (Figure 7A). To assess the diagnostic performance of the five models in the non-obese and obese MASLD subgroups, we applied each model to its respective subgroup. In the non-obese MASLD subgroup, the MASLD Score maintained high discriminatory power (AUC = 0.942), whereas the diagnostic validity of the other models was significantly lower (Figure 7B). In contrast, all models demonstrated robust diagnostic performance in the obese MASLD subgroup (Figure 7C; Table 5).

**TABLE 5:** Comparison of the diagnostic performance of the models in non-obese and obese MASLD populations.

Model	Group	AUC	Sensitivity (%)	Specificity (%)	PPV (%)	NPV (%)	PLR	NLR	Youden’s index
MASLD Score	Non-obese	0.942	88.1	86.3	88.9	85.3	6.43	0.14	0.744
	Obese	0.987	97.7	90.4	94.6	96.3	10.18	0.025	0.881
HSI	Non-obese	0.852	75.4	80	82.4	72.4	3.77	0.31	0.554
	Obese	0.964	95.3	90.4	93.7	92.7	9.92	0.052	0.857
K-NAFLD	Non-obese	0.868	86.4	71.6	79	81.2	3.04	0.19	0.58
	Obese	0.949	96.1	79.8	84.8	94.8	4.76	0.049	0.759
NSS	Non-obese	0.887	67.8	95.8	95.3	68.5	16.3	0.34	0.636
	Obese	0.972	96.1	88.3	90.8	95.2	8.23	0.044	0.844
NAFLD Ridge Score	Non-obese	0.663	59.3	72.6	68.4	64	2.16	0.56	0.319
	Obese	0.738	64.1	78.7	67.6	75.7	3.02	0.456	0.428

NPV, negative predictive value; PPV, positive predictive value; NLR, negative likelihood ratio; PLR, positive likelihood ratio.

## 4 Discussion

Research has demonstrated that the AGE/RAGE axis establishes a positive feedback loop that initiates a series of processes, including oxidative stress, inflammation, cellular malfunction, fibrosis, and apoptosis, all of which contribute to damage in end organs (18, 34). Conversely, the esRAGE provides a protective effect by inhibiting AGE/RAGE interactions (20, 26, 28). Therefore, a comprehensive assessment of AGEs and their receptors (esRAGE), is essential for understanding the development and progression of MASLD.

In the study, we discovered that the levels of AGEs were considerably elevated in MASLD patients than in healthy controls, aligning with findings from previous research. In a cross-sectional survey of MASLD cases (*n* = 67), serum AGEs were significantly elevated in both early and advanced stages of MASLD when compared to the normal group (25). Additionally, a controlled study of 58 MASLD patients and 58 healthy individuals found greater levels of late glycation markers, such as carboxymethyl-lysine, pentosidine, and AGE fluorescence, in the MASLD group (35). Interestingly, only three previous research have assessed the correlation between blood esRAGE levels and MASLD, despite the fact that several investigations have shown the link between esRAGE and liver disease. D’Adamo et al. (36) found that in a cohort of obese prepubertal children (*n* = 140, aged 6–10 years), serum esRAGE levels were lower in those with hepatic steatosis than in healthy controls. Francesca et al. (37) investigated 60 patients with MASLD and 50 without hepatic steatosis, finding that serum esRAGE levels were lower in the MASLD group. Conversely, Rohini et al. (38) reported that among 340 patients (BMI > 35) who underwent bariatric surgery, there were no significant differences in serum levels of esRAGE between those with MASLD and those without liver disease. These studies were conducted with smaller cohort samples or within specific populations and do not adequately reflect serum esRAGE levels in a broader MASLD population. Consequently, larger studies investigating esRAGE in MASLD are warranted. The present study was conducted in a larger cohort, serum esRAGE levels were elevated in patients with MASLD compared to healthy controls. Recent evidence suggests that RAGE activation leads to an increase in reactive oxygen species production and upregulation of various genes, including RAGE itself, thereby initiating a positive feedback loop (18). Since esRAGE is derived from RAGE (26), any alterations in RAGE levels are mirrored by changes in esRAGE levels. Consequently, the elevated RAGE levels are attributable to significantly increased serum AGEs, which may further elevate esRAGE levels. In conclusion, AGEs and esRAGE can be used as biomarkers for the diagnosis of MASLD.

The current study population found that the incidence was higher in females than males in non-obese MASLD populations. Recent studies have corroborated that non-obese MASLD is more prevalent among women (11, 39). Additionally, our findings revealed that non-obese MASLD patients were older than their obese counterparts, a result supported by a cross-sectional analysis of 946 MASLD cases (13). The comparison of serum AGEs and esRAGE levels between non-obese and obese MASLD patients found that serum AGEs were greater in obese MASLD patients, but there was no statistical difference. A comparison of correlations revealed a positive correlation between AGEs and BMI (*P* < 0.05). This was consistent with a bibliometric analysis that showed higher levels of serum AGEs in the presence of obesity (40). Notably, for the first time, our study discovered that obese MASLD patients had significantly elevated serum esRAGE levels than non-obese MASLD patients. Consistent with the idea in this paper, elevated amounts of AGEs can cause elevated levels of RAGE, which can then increase levels of esRAGE. There is a lack of studies on esRAGE in non-obese and obese MASLD, and the emergence of serum esRAGE as a non-invasive biomarker for non-obese and obese MASLD needs to be confirmed by further studies.

There have been numerous non-invasive and simple models created to identify MASLD (6, 7). The Fatty Liver Index (FLI), developed from a cohort of 5,780 individuals in Italy (10), is suitable for large-scale detection of MASLD (41). The Hepatic Steatosis Index (HSI) has demonstrated effectiveness as a simple screening tool for predicting MASLD in studies involving over 10,000 Korean patients (30). Other scoring systems, such as the Lipid Accumulation Product (LAP), can assess central lipid accumulation (42). MASLD results from a complex interaction of environmental, genetic, and dietary factors (43). Certain dietary practices, including excessive consumption of fructose and calories, as well as physical inactivity, are linked to the development of MASLD (44). However, there are notable differences between Asian and Western people’s food habits, lifestyles, and genetic origins (45). These scoring systems were originally designed for Western cohorts and may not be applicable to Asian individuals. Furthermore, there are notable variations in the clinical and metabolic features of individuals with MASLD who are not fat compared to their counterparts who are obese (13). Therefore, obesity should not be the only criterion for MASLD screening, particularly in light of the increasing prevalence of non-obese MASLD (12). This study developed a diagnostic model incorporating Age, WC, BMI, ALT, TG, HDL, AGEs, and esRAGE for diagnosing MASLD. To our knowledge, this is the first investigation to create a diagnostic model based on AGEs and esRAGE in MASLD patients. In this paper, we developed a nomogram to calculate the exact probability of having MASLD, facilitating the model’s application in clinical settings. The values of each variable in the nomogram are assigned scores on a scale from 0 to 100, and the scores are subsequently summed. The total sum is plotted on the total score axis, enabling the prediction of the probability of MASLD. Internal validation using Bootstrap resampling demonstrated good discrimination, indicating that the model we constructed is stable for prediction. The most crucial factor to consider when using a model is determining whether an individual requires further treatment or care based on their actual needs (46). To establish clinical utility, we used decision curve analysis to determine whether model-assisted decision-making would give clinical advantages to individuals. The decision curves indicated that using our model to predict MASLD could yield greater benefits compared to a “treat all” or “treat none” approach. In the present study, the MASLD Score demonstrated superior diagnostic performance compared with existing MASLD models (i.e., HSI, K-NAFLD, NSS, and NAFLD Ridge Score), achieving high accuracy in both the overall population and the non-obese subgroup, thereby overcoming the poor diagnostic performance of traditional models in non-obese MASLD patients. Therefore, this model might be a useful tool for Chinese community doctors in diagnosing MASLD.

Unavoidably, this study has certain limitations. First, due to the fact that this research is cross-sectional, we are unable to demonstrate that there is a causal association between the variables. Second, this study employed a single-center design with participants from a single region, which may introduce bias due to geographic diet, genetics, or environmental factors. Future multicenter studies are essential to validate the model’s generalizability, particularly by including cohorts from diverse ethnic, geographic, and socioeconomic backgrounds. In addition, although conventional ultrasound is widely employed for the clinical screening of MASLD, its capacity to quantitatively assess steatosis remains limited. Future studies should incorporate more precise imaging techniques (e.g., MRI-PDFF or controlled attenuation parameter) to further enhance diagnostic accuracy.

## 5 Conclusion

In conclusion, this cross-sectional study indicates that AGEs and esRAGE can be used as potential non-invasive biomarkers for differentiating patients with MASLD. Additionally, this study is the first to show that obese MASLD patients have considerably higher serum esRAGE levels than their non-obese counterparts. Finally, we create and evaluate a diagnostic model to predict the risk of MASLD, which assists in identifying high-risk individuals and enhancing strategies for the prevention and treatment of MASLD.

## Data Availability

The datasets presented in this study can be found in online repositories. The names of the repository/repositories and accession number(s) can be found in the article/supplementary material.
